# Capillary Regeneration in Scleroderma: Stem Cell Therapy Reverses Phenotype?

**DOI:** 10.1371/journal.pone.0001452

**Published:** 2008-01-16

**Authors:** Jo N. Fleming, Richard A. Nash, D. O. McLeod, David F. Fiorentino, Howard M. Shulman, M. Kari Connolly, Jerry A. Molitor, Gretchen Henstorf, Robert Lafyatis, David K. Pritchard, Lawrence D. Adams, Daniel E. Furst, Stephen M. Schwartz

**Affiliations:** 1 Department of Pathology, University of Washington, Seattle, Washington, United States of America; 2 Clinical Research Division, Fred Hutchinson Cancer Research Center, Seattle, Washington, United States of America; 3 Department of Dermatology, Stanford University School of Medicine, Stanford, California, United States of America; 4 Clinical Research Division, Pathology Section, Fred Hutchinson Cancer Research Center and Seattle Cancer Care Alliance, Seattle, Washington, United States of America; 5 Department of Medicine, University of California at San Francisco, San Francisco, California, United States of America; 6 Department of Rheumatology, Virginia Mason Medical Center, Seattle, Washington, United States of America; 7 Program in Transplantation Biology, Clinical Research Division, Fred Hutchinson Cancer Research Center, Seattle, Washington, United States of America; 8 Rheumatology Section, Boston University School of Medicine, Boston, Massachusetts, United States of America; 9 Department of Rheumatology, David Geffen School of Medicine, University of California at Los Angeles, Los Angeles, California, United States of America; Leiden University Medical Center, Netherlands

## Abstract

**Background:**

Scleroderma is an autoimmune disease with a characteristic vascular pathology. The vasculopathy associated with scleroderma is one of the major contributors to the clinical manifestations of the disease.

**Methodology/Principal Findings:**

We used immunohistochemical and mRNA in situ hybridization techniques to characterize this vasculopathy and showed with morphometry that scleroderma has true capillary rarefaction. We compared skin biopsies from 23 scleroderma patients and 24 normal controls and 7 scleroderma patients who had undergone high dose immunosuppressive therapy followed by autologous hematopoietic cell transplant. Along with the loss of capillaries there was a dramatic change in endothelial phenotype in the residual vessels. The molecules defining this phenotype are: vascular endothelial cadherin, a supposedly universal endothelial marker required for tube formation (lost in the scleroderma tissue), antiangiogenic interferon α (overexpressed in the scleroderma dermis) and *RGS5*, a signaling molecule whose expression coincides with the end of branching morphogenesis during development and tumor angiogenesis (also overexpressed in scleroderma skin. Following high dose immunosuppressive therapy, patients experienced clinical improvement and 5 of the 7 patients with scleroderma had increased capillary counts. It was also observed in the same 5 patients, that the interferon α and vascular endothelial cadherin had returned to normal as other clinical signs in the skin regressed, and in all 7 patients, *RGS5* had returned to normal.

**Conclusion/Significance:**

These data provide the first objective evidence for loss of vessels in scleroderma and show that this phenomenon is reversible. Coordinate changes in expression of three molecules already implicated in angiogenesis or anti-angiogenesis suggest that control of expression of these three molecules may be the underlying mechanism for at least the vascular component of this disease. Since rarefaction has been little studied, these data may have implications for other diseases characterized by loss of capillaries including hypertension, congestive heart failure and scar formation.

## Introduction

Progressive systemic sclerosis, or scleroderma, is an uncommon connective tissue disease characterized by autoimmunity, diffuse fibrosis[1] in the skin and internal organs, and a vasculopathy with intimal hyperplasia of muscular and elastic arteries[2], [3]. Raynaud's phenomenon, an excessive vasospastic reaction to cold or stress[4], precedes diagnosis of scleroderma by months or years[5], and is early evidence of the vasculopathy associated with scleroderma. The vasculopathy in scleroderma can result in non-healing ulcers, gangrene and digit loss[6], clinical features rarely encountered in primary Raynaud's disease.

Further evidence of endothelial damage in scleroderma are findings by nail fold capillaroscopy [7]. Typical findings include giant capillaries, hemorrhages, avascular areas and neovascularization[8]. This morphology resembles malformed capillary beds formed in response to vascular endothelial growth factor [9] and could be the result of increased levels of vascular endothelial growth factor produced in response to ischemia resulting from vasospasm and intimal hyperplasia[10]. Increased levels of vascular endothelial growth factor (VEGF) are known to be present in scleroderma[11] and high levels of VEGF are normally associated with increased angiogenesis. Instead there appears to be a loss of capillaries. Unfortunately, the prevailing belief that there is loss of capillaries in scleroderma is based on findings in nail fold capillaroscopy and is dependent on seeing columns of blood. The ischemia caused by Raynaud's phenomenon, especially coupled with malformations caused by high levels of VEGF could cause capillary collapse and could be misinterpreted as loss of capillaries since nail fold microscopy would be unable to identify unperfused capillaries. Definitive evidence for rarefaction, as opposed to loss of capillary blood flow or malformation of newly formed vessels, [8], [9], requires histological studies using endothelial specific markers[12].

Capillary rarefaction has been identified, to our knowledge, in only three other pathologic entities: congestive heart failure, granulation tissue and hypertension[13], [14]. Some researchers have suggested that rarefaction may contribute to the characteristic elevation in peripheral résistance in hypertension[14]. Scleroderma patients can also have cardiac failure associated with pulmonary fibrosis and pulmonary hypertension[15], [16] but a link has not been found between the rarefaction in scleroderma and that in cardiac failure, and scleroderma patients are, as a group, hypotensive[17] rather than hypertensive.

To investigate the vasculopathy associated with scleroderma we examined protein and mRNA molecules in endothelial cells and capillaries of skin biopsies from patients with diffuse cutaneous scleroderma and normal controls. In addition, we retrospectively studied skin biopsies from diffuse cutaneous scleroderma patients enrolled in a pilot study of the effect of high-dose immunosuppressive therapy and autologous hematopoietic cell transplantation (HDIT/HCT)[18]. After HDIT/HCT, these patients all experienced significant clinical improvement[19] including decrease in the dermal fibrosis[19], [20] and the modified Rodnan skin score (MRSS) and an increase in overall function as measured by the modified Health Assessment Questionnaire Disability Index (MHAQ) . We analyzed biopsies from these patients, both before and after treatment, and have demonstrated a correlation between changes in tissue expression of angiogenesis related molecules and capillary numbers.

## Results

### Scleroderma vessels lost normal endothelial markers and gained inflammatory markers

Normal dermal endothelium is characterized by several characteristic immunohistochemical markers including: CD31, a common blood group antigen (*Ulex europaeus* lectin), von Willebrand factor, and VE cadherin[21]. The most widely used of these is CD31, which also stains leukocytes[22]. We found the expected vascular labeling of vessels in normal and scleroderma skin, with some positive leukocytes. Endothelium in capillary loops and precapillary arterioles in normal skin are also positive for enzymatic activity of endogenous alkaline phosphatase[23].

**Figure 1:**
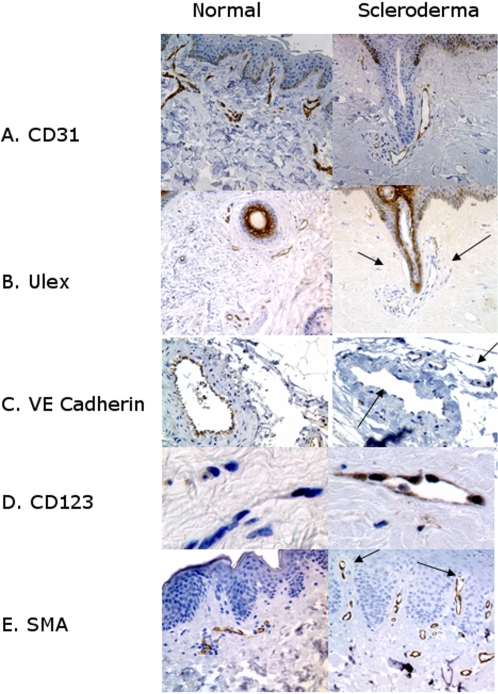
Endothelial Phenotype of Capillaries in Scleroderma Compared to Normal Controls. Markers of normal endothelium: A.) CD31 staining highlights capillaries in both scleroderma and normal controls. B.) Black arrows show *Ulex europaeus* lectin (Ulex) is lost from some vessels in scleroderma (compare with CD31 above). C.) VE Cadherin stain is lost from some vessels in scleroderma (arrows) whereas all vessels in normal skin are positive for VE cadherin. Both von Willebrand factor and alkaline phosphatase were similarly lost from scleroderma were similarly lost from scleroderma (data not shown) Markers of inflamed endothelium: D.) CD123, a marker for high endothelial venules, is increased in scleroderma as is E.) smooth muscle actin (SMA). In the capillaries (arrows) smooth muscle actin stain extends to the very top of the dermal papilla, unlike normal skin where smooth muscle actin stain ends at the superficial horizontal plexus (doubleheaded arrow). SMA staining is also increased in the media of vessels in the reticular dermis, (data not shown)

Figure 1 (A and B) depicts normal and scleroderma biopsies where serial sections have been stained with CD31 and *Ulex europaeus* lectin. In scleroderma skin a population of vessels have no luminal staining for *Ulex europaeus* lectin whereas normal skin has robust staining in all vessels. Von Willebrand factor, VE cadherin (Figure 1 C), and alkaline phosphatase all showed similar marked loss of luminal stain.

Immunohistochemical and histochemical stain quantification showed significant loss in vessels in scleroderma biopsies for *Ulex europaeus* lectin (p = 0.01), alkaline phosphatase (p = 0.002), VE cadherin (p = 0.008), and von Willebrand factor (p = 0.033) (Table 1 Section A). The difference in sample numbers tested for each marker is a function of scarcity of tissue and when the IHC was done. Some samples were very small and IHC was done for only a few markers. Endothelial markers were scored according to presence of positive and negative vessels. Numbers of biopsies with vessels that all had stain on the endothelial cells are expressed as number of positive biopsies per total n. All endothelial markers showed a decrease in expression in scleroderma except CD31.

**Table 1:** Molecular markers of microvascular phenotype, inflammation and cell cycle in skin biopsies from scleroderma compared to controls.

Type of molecular marker	Normal #pos/n†	Scleroderma #pos/n†	P value ‡
**A) Endothelial markers**			
Alkaline Phosphatase	4/4	0/8	0.002
VE Cadherin	4/6	0/10	0.008
CD31	13/13	13/13	1
Von Willebrand Factor	3/3	1/7	0.033
*Ulex Europaeus* Lectin	10/14	3/14	0.01
**B. Inflammatory endothelial markers**			
CD123 (high endothelial venules)	1/12	12/16	0.0005
VCAM1	0/4	5/5	0.007
ICAM1	0/3	5/5	0.017
CD62P	0/7	1/7	0.49
Smooth muscle actin	0/15	12/12	0.000001
**C. Other inflammatory markers**			
PAR2	0/4	5/5	0.007
PSGL1	0/7	8/8	0.0001
IL-1 α	0/6	7/7	0.0005
**D. Other markers**			
*RGS5 in situ*	0/5	7/8	0.004
Interferon α *in situ* (*IFNA1* and *IFNA2*)	0/6	9/9	0.0001
CD123 (plasmacytoid dendrtic cells)	1/12	14/16	0.000005
STAT1	0/7	7/10	0.006
**E. Endothelial cell death (apoptosis)**			
Cleaved Caspase 3	0/7	0/17	1
**F. Perivascular cell Turnover:**			
Ki67 Antigen	0/8	11/11	0.00001
**G. Endothelial cell turnover**			
Ki67 antigen	0/7	0/17	1

† Data are numbers biopsies with positive immunohistochemical, lectin and *in situ* staining of skin biopsies over total number of biopsies available for staining.

‡ P values were calculated with Fisher's exact test using 2×2 frequency tables.

We assessed the vasculature for signs of endothelial inflammation and summarized the quantitative results in Table 1 B. High endothelial venules have been described in dermal vessels of chronic dermopathies and are considered a sign of chronic inflammation[24]. The high endothelial venule phenotype has endothelial nuclei projecting into the lumen with characteristic proteins on the endothelial surface that provide a specialized site for lymphocyte migration [25]. CD123, CD62P, and smooth muscle actin are among the proteins expressed on inflamed vascular lumens[26], [27]. Scleroderma patients clearly had increased inflammatory markers of endothelial cells compared to normal controls. The endothelial cells in scleroderma were found to have significantly increased expression of CD123 (high endothelial venule, p = 0.0005) and smooth muscle actin (p = 0.000001) although CD62P did not show a significant difference (p = 0.49). CD123+ (high endothelial venules), and smooth muscle actin staining patterns are depicted in Figure 1 D and E respectively.

Other markers of inflammation not limited to endothelial cells were significantly increased in scleroderma compared to normal tissue (quantitative results summarized in Table 1C). Immunohistochemical stains for VCAM1 and ICAM1 were elevated and statistically significant (p<0.05), consistent with published data for higher expression in scleroderma compared to normal[28], and cells positive for p selectin glycoprotein ligand 1(p = 0.0001), interleukin-1α (p = 0.0005) and protease activated receptor 2 (p = 0.007) were all increased in scleroderma compared to normal controls (quantitative results summarized in table 1 C). PAR2 appeared concentrated in smooth muscle and endothelial cells, with some positive cells scattered in the dermis biopsies (photomicrographs not shown). PAR2 is expressed in response to inflammatory stimuli and in turn appears to enhance endothelial proliferation, angiogenesis and immune mediator production resulting in a neurologically mediated itch [29]–[31], a clinical phenomenon seen often in early diffuse cutaneous scleroderma [30], [32]–[34]. IL-1α and PSGL1 were similarly increased both in endothelial cells and in cells present in the dermal microvascular unit (photomicrographs not shown). IL-1α activates complex pathways in endothelial cells, dramatically affecting function. PSGL1 is the high affinity counter receptor for selectins and is constitutively expressed on some kinds of inflamed endothelium [35]and PSGL1-selectin interactions play a role in homing and entry of inflammatory cells into sites of inflammation[36].

### Scleroderma skin had an infiltrate of CD123+cells, an increase in interferon α mRNA expression and downstream signaling markers of interferon

**Figure 2:**
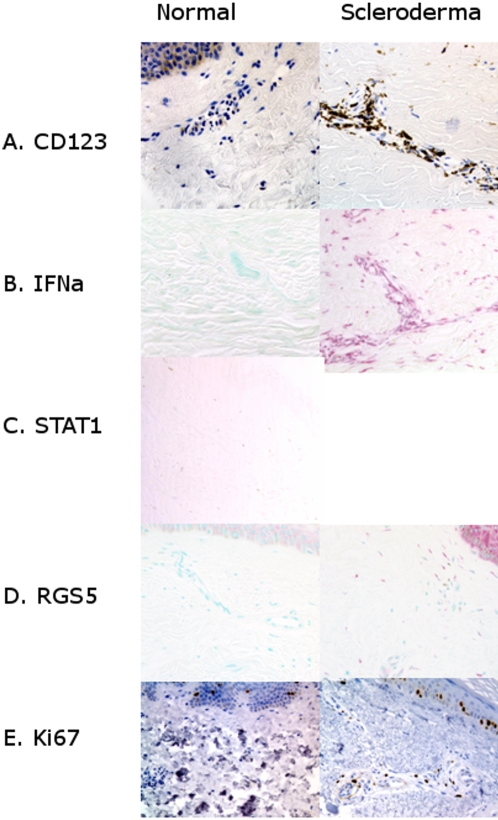
Non-Angiogenic Phenotype in Scleroderma Compared to Normal Controls. A) There was increased staining in CD123 in perivascular cells in the superficial horizontal plexus in skin biopsies from scleroderma patients compared with normal controls. B) Interferon a (IFNa) *in situ* hybridization exhibited a robust signal in scleroderma but was absent from controls. C.) Nuclear staining of phosphorylated STAT1 in scleroderma provided evidence of active interferon signaling, with STAT1 phosphorylation, dimerization and translocation to nucleus in response to interferon a. D.) *RGS5*, a marker of pericytes and arterial smooth muscle had increased mRNA expression in scleroderma in a perivascular distribution. E.) Ki67 a marker of cell cycle, showed no sign of Ki67 labeling of the endothelial cells in either normal controls or scleroderma. There was an increase in Ki67 positive perivascular cells in scleroderma.

While looking at high endothelial venules in scleroderma skin (above), we found a population of strongly CD123+ cells infiltrating the dermis. Scleroderma dermis had many CD123+cells in the superficial horizontal plexus, significantly more than normal skin (p = 0.000005, Table 1D). CD123+ cells known as plasmacytoid dendritic cells are the primary producers of interferon α in the human body[37]. To look for the presence of interferon α mRNA expression we used *IFNA1* and *IFNA2* RNA *in situ* probes to look for expression of interferon α mRNA. 100% of Scleroderma patients were positive for interferon α and 0% of the normal had interferon α (p = 0.0001). Normal skin showed no detectable hybridization in the dermis and occasional faint hybridization in the epidermis whereas scleroderma tissue always expressed interferon α mRNA in the dermis and epidermis. The interferon α+cells, probably plasmacytoid dendritic cells, were present next to small vessels in the superficial horizontal plexus in the upper dermis (Figure 2 B). In situ results were repeated 3 times and results were consistent.

To further confirm the presence of interferon α, we used staining for downstream markers of type 1 interferon activity. Canonical type 1 interferon signaling involves the binding of type 1 interferon (interferon α β or ω) to the type 1 interferon receptor, activation of the JAK/STAT pathway, and phosphorylation of STAT1[38]. Presence of phosphorylated STAT1 in the nucleus of cells STAT1 dimerization with another phosphorylated STAT and translocation into the nucleus. STAT1 phosphorylation was present in cellular nuclei in 7 of 10 scleroderma biopsies (depicted in Figure 2 C), but negative in all 7 normal controls tested (p = 0.006, Table 1 D).

### Scleroderma skin had increased mRNA expression of RGS5 and no sign of endothelial death or proliferation

The apparent loss of capillaries in scleroderma led us to look for increased expression of *RGS5* mRNA. We found that both *RGS5* mRNA expression was significantly increased in scleroderma (Table 1 D). *RGS5* is a regulator of G protein coupled receptor signaling with cardiovascular properties[39] and specific association with arteries[40]. For as yet unknown reasons, *RGS5* is expressed in pericytes that coat angiogenic vessels and appearance of this gene correlates with loss of the ability to branch[41]. *RGS5 in situ* probes showed <1*RGS5*+cells in normal skin. The same probes hybridized with >10 cells per hpf cells in the 7 of 8 biopsies of scleroderma skin, both in a perivascular distribution, and scattered throughout the dermal matrix in cells that seem to be myofibroblasts. There was a strong signal in the epidermis of the scleroderma patient as well (Figure 2 D).

For the same reasons we looked at *RGS5* we looked for direct evidence of endothelial cell death and/or signs that vessels were growing to replace the lost capillaries. To assess for signs of apoptosis, the tissue was examined for nuclear changes characteristic of apoptosis and stained for the apoptotic marker cleaved caspase 3 [42] (Table 1 E). Cleaved Caspase 3 staining is recorded as positive biopsies per total n. Numbers of caspase 3+cells per high power field in the scleroderma biopsies fell within the normal range. The only positive biopsy was one of the normal controls (photomicrograph not shown) and the quantitative results were not significantly different (p = 1). P values were calculated with Fisher's exact test using 2×2 frequency tables.

To assess endothelial replication, positive Ki67 antigen staining was used to indicate the presence of cells in the biopsy that were in the cell cycle, providing an estimate of proliferation. Ki67 staining is expressed as number of positive biopsies per total n. Cells in the follicles and epidermal layer were not counted. All biopsies were scanned for cells lining the lumen that labeled positive with Ki67 and none were found. We scored the presence of Ki67+cells in normal and scleroderma biopsies and carefully assessed cellular morphology to establish whether or not endothelial cells were proliferating (Table 1 F and G.). Comparison of Ki67 stain between scleroderma and normal skin biopsies demonstrated no endothelial cells in cell cycle in the superficial horizontal plexus or anywhere else in any of the biopsies of normal or scleroderma skin (Figure 2 E). Ki67+cells were found only in a perivascular distribution. With no evidence that endothelial cells are proliferating in the scleroderma biopsies, we suggest that there is no ongoing endothelial cell replacement.

### Scleroderma had a decreased numbers of capillaries

**Figure 3:**
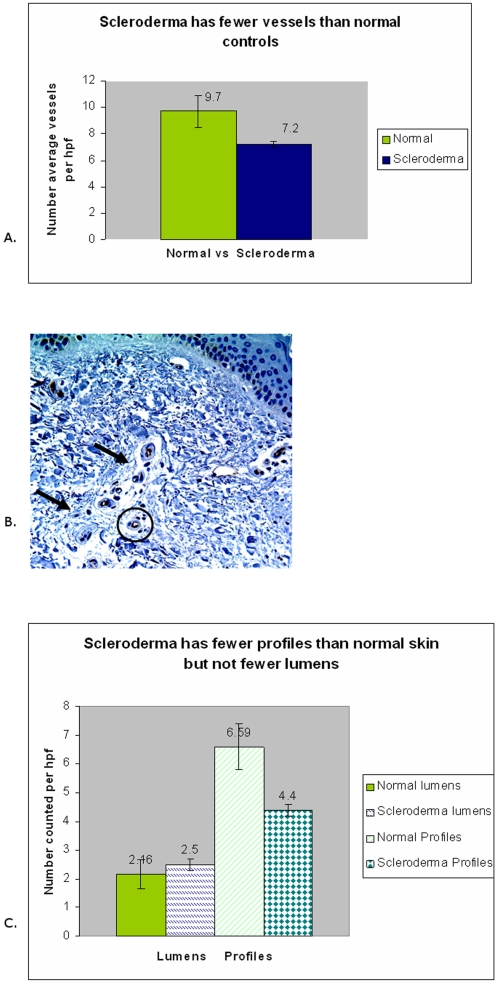
Total Vessels Were Decreased and Capillaries in Scleroderma Skin Were Selectively Decreased. A.) This data represents the total number of vessels per hpf in 15 normal and 21 scleroderma skin biopsies stained with CD31. The average vessels/hpf is significantly decreased in scleroderma compared with normal controls (p = 0.02). B.) Normal skin IHC for CD31 demonstrates microvasculature. “Profiles” or capillaries are defined as linear CD31 staining with no central lumen (single arrows) and represent a robust estimate of capillary density. ‘Lumens’ represent larger vessels, and are defined as structures positive for CD31 that have a central lumen (circle). C.) Data are average numbers of “lumens” versus “profiles”. Average “lumens”, representing larger vessels, in scleroderma skin biopsies are not significantly different from normal skin (p = 0.18). Average “profiles”, representing capillaries, are significantly decreased in scleroderma compared with controls P = 0.009. These results indicate that capillaries are decreased in scleroderma.

Finally, to confirm that rarefaction exists in scleroderma, we quantified the number of vessels present in normal and scleroderma skin biopsies. Vessel counts based on finding red blood cells can be misleading when vessels are collapsed; therefore we used the presence of CD31 to define vascular profiles. We compared average numbers of vessels per high power field between forearm skin sections from 21 scleroderma patients and 13 site-matched normal controls. To determine if blood vessels decrease during the course of disease, we counted biopsies from our patient group whose disease duration was 2 years or less, and compared vessel density to biopsies whose disease duration was five years or greater and compared both disease groups to normal vessel counts (Figure 3A). The numbers of vessels per high power field in scleroderma was virtually identical both early (2 years or less) and late in disease (5 or more years) and there was no statistically significant difference (p = 0.48). Vessel counts of normal controls differed significantly compared with both early (p = 0.02) and late (p = 0.04) scleroderma and showed that the number of total vessels was decreased overall in scleroderma (Figure 3 A). To approximate capillary numbers, *profiles* of vessels were counted in skin sections stained with an antibody for CD31. *Profiles* were defined as clusters of positive CD31 stained cells within the dermis with no central lumen visible (Figure 3B). To approximate the numbers of larger vessels and the remainder of capillaries we counted *lumens* in the same sections. *Lumens* were defined as clusters of CD31 stained cells with a central lumen clearly visible (Figure 3B). Comparison of *lumens* and *profiles* between controls and scleroderma (Figure 3C) show that *profiles* were significantly decreased in scleroderma (p = 0.009) whereas *lumens* were not. (p = 0.18). This suggests that vascular loss in early diffuse scleroderma is in smaller vessels, likely representing capillaries.

### Scleroderma endothelia regained a normal endothelial marker and lost inflammatory markers after HDIT/HCT treatment

Next we evaluated biopsies from patients with scleroderma who underwent HDIT/HCT. Our study of capillary counts, IHC and in situ study included biopsies from 7 patients at baseline and at a median of 5 (range 4–6) years after treatment. As noted in table 2, the patients from the clinical trial whose skin biopsies were evaluated for the effects of HDIT/HCT on the microvasculature, all experienced improvement in clinical assessment of scleroderma (results of clinical trial have been previously published)[19], including activities of daily life, stamina, energy, and shortness of breath. Each of the 7 patients also reported improvement in range of motion, hand flexion and reduction in skin tightening, and pathologically the degree of the dermal fibrosis improved [19].

**Table 2:** Clinical and pathological findings after HDIT and HCT correlated with vessel counts.

Patient #	Specimen Code #	Time after HDIT/HCT (years)	Before HDIT/HCT vessels count	After HDIT/HCT Vessels count	Drop in MRSS after HDIT/HCT	Drop in dermal fibrosis After HDIT/HCT	Change in MHAQ score After HDIT/HCT
9	0942	6	6.8	7.7	16	3	1 to 0
11†	1105	5	6.14	7.9	30	5	2.5 to 0
12‡,Δ	1226	1	8.2		8	1	0.75 to 0
18	1888	5	5.2	12	18	3	2.125 to 1.5
**22** €,£	**2218**	**4**	**6.59**	**3.43**	**22**	**4**	
24	2435	4	5.09	8.78	6	4	2.875 to 2.375
**26** £	**2622**	**4**	**5.7**	**4.4**	**34**	**4**	**2.875 to 0.125**

For each of the clinical parameters assessed, post-HDIT/HCT values were compared to pre-HDIT/HCT (baseline) values at the last time point in each of several post-HDIT/HCT time windows

† Reported finger contractures as the only indication of evidence of scleroderma post HDIT/HCT.

‡ No clinical or pathologic data is available for the first 4 years post HDIT/HCT. Patient subsequently underwent right lower lobectomy 4 years 9 months post HDIT for squamous cell carcinoma.

€ developed pulmonary hypertension.

£ did not have increased capillary counts in post HDIT/HCT biopsies.

Δ did not have a count done on the one year biopsy and clinical data was not available for four year biopsy

Abbreviations: mRSS-Modified Rodnan Skin Score for dermal fibrosis; MHAQ–modified Health Assessment Questionnaire Disability Index

**Figure 4:**
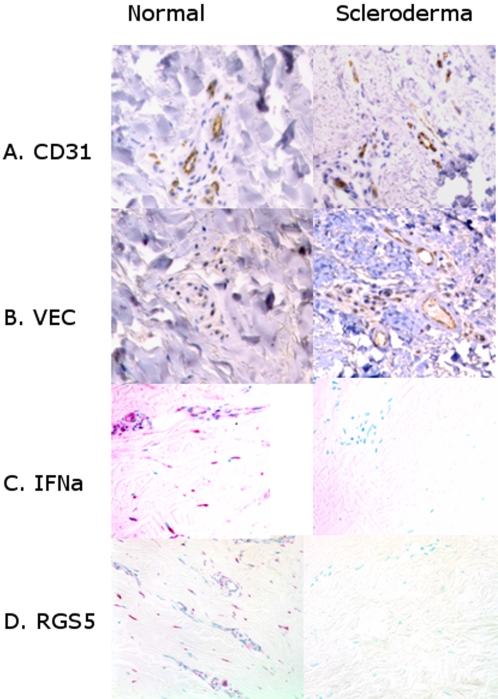
Immunohistochemistry and in situ results before and after HDIT/HCT show regain of VE cadherin and loss of RGS5 and Interferon α. A.) Immunohistochemistry for CD31 before and after HDIT and autologous HCT is positive in both biopsies, whereas B) when compared with CD31 stained biopsies above, VE cadherin is negative before but positive after HDIT and HCT. C.). Baseline biopsies of scleroderma patients prior to HDIT and autologous HCT all showed cells positive for interferon α (IFNa) *in situ* hybridization and were present both in the vasculature and scattered in the matrix. After HDIT and autologous HCT, interferon α mRNA was undetectable in 5 of 7 biopsies. Compared biopsies were run at the same time with the same conditions and probe concentration. D) *In situ* hybridization for *RGS5* demonstrates positive mononuclear cells around vessels and some cells scattered in the matrix in the scleroderma biopsies prior to treatment with HDIT/HCT. There was little or no detectable *RGS5* mRNA after HDIT/HCT.

We looked at endothelial phenotype, comparing endothelial marker expression by IHC, and found that alkaline phosphatase, *Ulex europaeus* lectin, and von Willebrand factor did not return to normal after HDIT/HCT. CD31 was consistently positive both before and after HDIT/HCT. VE cadherin stain was absent in a population of vessels in all baseline biopsies. No VE cadherin-negative vessels were present in 5 of 7 biopsies after HDIT/HCT (p = 0.01, Table 3A, see photomicrographs figure 4A and B for CD31 and VE cadherin).

**Table 3:** Molecular markers of microvascular phenotype, inflammation and cell cycle in skin biopsies from scleroderma before and after HDIT/HCT

Type of molecular marker	Before HDIT/HCT # pos/n†	After HDIT/HCT # pos/n†	P value‡
**A) Endothelial markers**			
Alkaline Phosphatase	0/7	0/7	1
VE Cadherin	0/7	5/7	0.010
CD31	6/7	7/7	0.49
Von Willebrand Factor	1/7	4/7	0.13
*Ulex Europaeus* Lectin	2/7	3/7	0.49
**B Inflammatory**			
CD123 (high endothelial venules)	6/7	1/7	0.01
Smooth muscle actin	5/5	2/5	0.08
**C. Other :**			
*RGS5 in situ*	6/7	7/7	0.0023
Interferon α *in situ* (*IFNA1* and *IFNA2*)	7/7	2/7	0.01
STAT1	6/6	0/6	0.001
CD123 (plasmacytoid dendritic cells)	6/7	1/7	0.01
**D. Perivascular cell Turnover:**			
Ki67 Antigen	7/7	5/7	0.01
**E Endothelial cell turnover**			
Ki67 antigen	0/7	0/7	1
Cleaved Caspase 3	0/7	0/7	1

† Data are numbers of biopsies with positive immunohistochemical, lectin and *in situ* staining of skin biopsies over total number of biopsies available for staining.

‡ P values were calculated with Fisher's exact test using 2×2 frequency tables.

CD123+(high endothelial venules) endothelial cells significantly decreased (p = 0.01) after HDIT/HCT in scleroderma patients, although smooth muscle actin expression was not (p = 0.08).

### Interferon α mRNA expression in scleroderma decreased after HDIT/HCT treatment

Interferon α mRNA *in situ* hybridization, CD123 (plasmacytoid dendritic cells) and STAT1 were all significantly decreased after HDIT/HCT (Table 3C). At baseline, all 7 patients had markedly high transcript levels of interferon α RNA both in the dermis and epidermis whereas five of 7 patients after HDIT/HCT showed loss of RNA hybridization for interferon α in the dermis and epidermis (p = 0.01, see photomicrographs Figure 4C). The marker for plasmacytoid dendritic cells, CD123, present in all baseline biopsies preceding HDIT/HCT, was lost in 6 of 7 patients after HDIT/HCT (p = 0.01) To further confirm the loss of interferon α expression, presence of nuclear phosphorylated STAT1 was decreased (p = 0.001) after HDIT/HCT (Table 3B). The decrease in signs of interferon α, CD123+plasmacytoid dendritic cells and STAT1 may indicate that the interferon α producing cells are destroyed or deactivated by treatment, and in the context of the capillary counts below have interesting vascular implications.

### After HDIT/HCT scleroderma skin had decreased mRNA expression of RGS5 and no sign of endothelial death or proliferation

RNA *in situ* hybridization for *RGS5* at baseline shows that 6 of 7 scleroderma patients had increased positive cells (p = 0.0023, Table 3C). After HDIT/HCT, all 7 biopsies had less than one per high power field (depicted in Figure 4D).

Biopsies before and after had numbers of perivascular cells in cell cycle greater than normal (p = 0.09, Table 3D). Ki67 was not seen in endothelial cells before and after HDIT/HCT (p = 1, Table 3E). IHC for cleavage of caspase 3 and histological analysis of the endothelial cells before and after HDIT/HCT showed very little cell death (p = 1, Table 3F).

### After HDIT/HCT, capillary numbers increased in those patients who lost all interferon α expression, and regained VE cadherin

**Figure 5:**
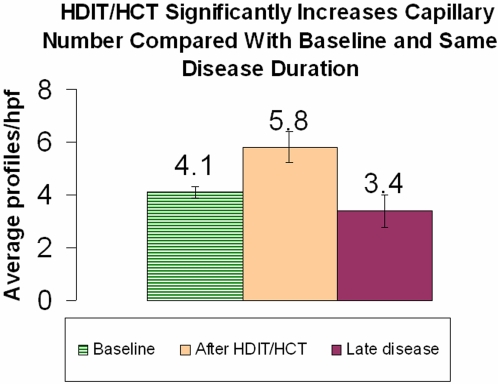
Capillary Counts Increase After HDIT and HCT Compared to Baseline and to Scleroderma of Long Duration. Data are average “profiles”/hpf counted in scleroderma patients before (Baseline) and after (After HDIT/HCT) treatment with HDIT and HCT. “Late disease” are average “profiles”/hpf in a separate group of patients with disease duration similar to patients after HDIT/HCT, included to provide an additional disease control. Baseline (biopsies for patients who grew back capillaries) had an average capillary number of 4.1/hpf. After HDIT/HCT, average capillary number (for patients who increased capillary number) rose to 5.8/hpf (p = 0.015). Patients with a disease duration of 5 or more years averaged 3.8 profiles/hpf and when compared with the patients who grew back capillaries(After HDIT/HCT) yielded a p value of 0.025.

To demonstrate whether capillary counts increase after treatment, we quantified biopsies from the scleroderma patients who grew back capillaries at baseline and after HDIT/HCT. Patients after HDIT/HCT show significantly increasing capillary numbers (p = 0.015, Figure 5). Most interesting, because of the mechanistic implications, is the correlation of the capillary numbers with VE cadherin and interferon α expression. All 7 patients showed changes in interferon α and VE cadherin in baseline biopsies. In the 5 patients where capillary counts increased, IHC for VE cadherin was positive in all vessels (Table 4). In the same patients where VE cadherin expression returned to normal, *IFNA1* and *IFNA2 in situ* hybridization (interferon α) was undetectable (Table 4, see Figure 4 C for photomicrographs).

**Table 4:** Increased vessel number following HDIT/HCT is correlated with return of normal immunohistochemical expression of VE cadherin and α interferon in skin biopsies in late stage scleroderma

Patient #	Change in Average vessel/hpf	Time after HDIT/HCT (years)	Before HDIT/HCT VE cadherin	Before HDIT/HCT interferon α	After HDIT/HCT VE cadherin	After HDIT/HCT interferon α
9	+0.9	6	neg	pos	pos	neg
11	+1.8	5	neg	pos	pos	neg
12	+2.6	4	neg	pos	pos	neg
18	+7	5	neg	pos	pos	neg
**22** †	**−3.2**	**4**	**neg**	**pos**	**neg**	**pos**
24	+3.7	4	neg	pos	pos	neg
**26** †	**−1.3**	**4**	**neg**	**pos**	**neg**	**pos**

† these 2 patient's skin biopsies had decreased numbers of vessels

### Late stage scleroderma did not resemble scleroderma after treatment

Skin changes in scleroderma at a late stage of disease can include some softening and reduction of symptoms[43], [44]. These late stage skin changes could be a potentially confounding factor for the scleroderma patients after HDIT/HCT since their disease duration spans 5 to 7 years after diagnosis. We therefore included as a final control, 4 skin biopsies from patients with a similar duration of disease (5–6 years from diagnosis). These biopsies were compared to the biopsies of the 7 patients after HDIT/HCT.

We compared the IHC results after HDIT/HCT with four patients whose disease duration is 5 years or longer. VE cadherin was negative in scleroderma of long duration, 4 of 4 Non-HDIT/HCT scleroderma patients of 5 years or greater scleroderma duration still have vessels that do not express VE cadherin. 4 or more years after HDIT/HCT however, 5 of 7 patients have regained all VE cadherin expression in biopsies (p = 0.007, Table 5 A). VE cadherin expression has therefore returned only after HDIT and is not an artifact of late stage disease.

**Table 5:** Scleroderma Patients after HDIT/HCT with Capillary Regeneration, Compared with Patients of Similar Disease Duration

Type of molecular marker	After HDIT/HCT # pos/n†	Same disease duration # pos/n†	P value‡
**A) Endothelial Markers**			
CD31	7/7	4/4	1
VE Cadherin	5/7	0/4	0.007
**B Inflammatory markers**			
CD123 (high endothelial venules)	0/5	3/3	0.017
**C. Other Markers**			
Interferon α *in situ* (*IFNA1* and *IFNA2)*	0/5	2/2	0.047
CD123 (plasmacytoid dendritic cells)	0/5	3/3	0.017

† Data are numbers of biopsies with positive immunohistochemical, lectin and *in situ* staining of skin biopsies over total number of biopsies available for staining.

‡ P values were calculated with Fisher's exact test using 2×2 frequency tables.

The CD123+endothelial cells (high endothelial venules) has resolved itself in 6 of 7 biopsies after HDIT/HCT, whereas Non-HDIT/HCT patients with similar (5 years or greater) disease duration all have CD123+ vessels with high endothelial morphology (p = 0.017, Table 5B). Similarly the expression of interferon α mRNA by i*n situ* hybridization is undetectable in 5 of 7 biopsies, whereas 2of four late stage disease patients have interferon α+ cells in dermis (p = 0.047, Table 5C). The perivascular cells positive for CD123 are also increased in late stage patients but have disappeared in all but one of the post HDIT/HCT biopsies (p = 0.017, Table 5C). Loss of the expression of interferon α, absence of CD123+cells, as well as inflammation, appear also not to be an artifact of late stage disease but seem to be associated with HDIT/HCT. Limited amounts of available tissue prohibited us from obtaining results for *RGS5.*

Profile counts showed a significant increase (p = 0.025) in the average number of capillaries per hpf in the five patients who grew back capillaries after HDIT/HCT, compared with Non-HDIT/HCT scleroderma of 5–6 years duration (Figure 5).

## Discussion

In this study, the most impressive changes in the dermis of scleroderma patients were rarefaction of capillary loops, loss of expression of VE cadherin, and the appearance of mRNA for interferon α and *RGS5*. While we also observed changes in other molecules, the changes in these three molecules are particularly relevant because they have all been reported to play possible critical roles in angiogenesis. We also report that after HDIT/HCT the above changes reverse, especially when increasing capillary counts are found in the tissue. We showed that these changes are unlikely to be the result of the natural history of scleroderma, and suggest that these findings may provide clues to the pathogenesis of this poorly understood disease.

An endothelial phenotype that lacks normal markers is a puzzle and to the best of our knowledge this is previously unreported. One extreme possibility is that endothelial cells no longer line the vessels. We do not think this is true. On two samples of normal and scleroderma skin, we performed electron microscopy (data not shown). The luminal cells of these samples lacked features of other cell types, which can, under some circumstances, form a lining. For example, we looked for the microplicated surface of macrophage or the altered polarity and microfilaments characteristic of smooth muscle cells and found none of these characteristics in the luminal cells in scleroderma vessels. One could speculate a variety of mechanisms to explain endothelial cells without normal markers: 1) differentiation of a cell type other than normal dermal microvascular endothelial cells into an endothelial-like morphology, which replaced the dead endothelial cells early in disease; 2) circulating precursors from other endothelial lineages (e.g. sinus, lymphatic) replace the normal dermal endothelial cell; 3) activating autoantibodies to endothelial cells or other circulating factors, which cause them to differentiate into an abnormal state and/or cause chronic inflammation; 4) effect of a pathological cell type on the endothelial cell expressome, or 5) an underlying mechanism or structural abnormality existent in scleroderma endothelial cells responsible for some part of the vascular abnormality similar to that found in the children of patients with malignant hypertension[45].

The bulk of capillaries in the skin are capillary loops branching from the superficial horizontal plexus into the rete pegs and consist of a single layer of endothelium with a few pericytes all above the border of the rete ridge[46]. This is a highly specific structure, optimized to improve thermal exchange. Our data showed that capillary loops, are lost from scleroderma skin at a very early stage. This loss was associated with a dramatic and unusual loss of expression of markers used to define the endothelial cell lineage including VE cadherin. We found significant loss of the smallest vessels in the baseline scleroderma skin biopsies (all 2 years or less from diagnosis). The low number of vessels appeared to remain constant at all stages of disease. After HDIT/HCT the endothelial marker which did return to normal in 5 of 7 patients, VE cadherin, is a calcium dependent cell adherens junction protein responsible during development for aggregation of cells and formation of endothelial tubes[47]. The loss of VE Cadherin in baseline scleroderma and the reappearance of VE cadherin after HDIT/HCT may be significant in terms of the angiogenic potential of the skin, since the same 5 patients whose VE cadherin expression returned to normal also increased the numbers of capillaries present in their skin.

*RGS5* mRNA expression was reversed in all 7 patients after treatment, and did not correlate specifically with regeneration of capillaries, since two of the seven did not increase average skin capillary numbers. This indicate that decreased *RGS5* expression after HDIT/HCT is insufficient to regenerate capillaries by itself, but like the VE Cadherin data may suggest that some form of vasostatic process in scleroderma inhibited any angiogenic response prior to treatment. The hypothesis exists that part of pathogenesis of scleroderma is circulation of an endothelial toxic agent, loss of circulating endothelial precursors or some other less specified dysregulation of the angiogenic pathway[48], [49]. How this relates to the loss of VE cadherin and the appearance of *RGS5* is not at all obvious.

Interferon α is a cytokine known to be a potent inhibitor of angiogenesis[50], [51]. Type 1 interferon has been found to be present in epithelial layers and epidermis in humans and identified as an endogenous inhibitor of angiogenesis in skin[52]. Interferon α treatment has been associated with new onset or exacerbation of scleroderma and Raynaud's phenomena with digital gangrene[53]–[55]. A type 1 interferon gene expression signature has been described in autoimmune connective tissues diseases, including scleroderma[56]–[58]. We found CD123+ interferon-producing cells[59] at the superficial horizontal plexus where the bulk of capillaries would be, and where angiogenesis should be taking place. Furthermore, increased STAT1 expression in the same areas confirms the presence of an interferon response. The evidence that expression of interferon α mRNA was decreased 5 of 7 patients undergoing HDIT/HCT combined with the unexpected observation of vascular regeneration suggest that an antiangiogenic state may have been reversed by this treatment. We are unaware of any other study that has shown reversible rarefaction in any disease. Intriguingly, the two patients interferon α expression was still present were the same two patients that did not grow new capillaries and had vessels negative for VE Cadherin.

Existing observations suggest that vascular disease is paramount in the pathogenesis of scleroderma[60], [61]. Certainly vasospasm, as in Raynaud's, is a frequent, though not a clearly obligatory precursor to disease. It is also possible that rarefaction precedes diagnosis of scleroderma by many years and our data suggest this may be the case. There are no objective data of which we know showing elevated levels of cell death or apoptosis in any phase of this disease. Suggestions that apoptosis and rarefaction occur during active disease have been based on studies showing the presence of circulating factors able to kill endothelial cells in culture[62] but the relevance to endothelial death in vivo is not known. Similarly, endothelial autoantibodies in scleroderma have been classified as anti-microvascular and are cited by some as a cause of vessel loss[63], [64]. Once again, these data are limited to *in vitro* studies that may not represent *in vivo* phenomena. Our studies were of skin biopsies only and, especially in the context of the HDIT/HCT trial, fresh tissue samples were limited. Given the number of possible circulating factors which may cause the death of endothelial cells or prevent their growth, it may be best in future studies to approach this problem with a discovery biology approach (such as proteomics or gene expression arrays) rather than a direct method. Also, since the natural history of the disease tends to decrease the levels of inflammatory factors as the disease moves from an inflammatory to a chronic sclerotic phase, it may not be possible to find significant differences without much larger numbers of patients than we have in this study. A larger study is underway and, given the clinical success of this one, and we may be able to assess antiendothelial antibodies and other circulating factors together with a more detailed assessment of cell death. We found no evidence of ongoing endothelial cell death in the scleroderma skin compared with normal controls, and must conclude that any endothelial cell death in scleroderma, implied by capillary rarefaction, must be an early or episodic event in scleroderma. To further support the suggestion that capillaries are lost very early, we found no evidence that capillary numbers continue to drop over time in scleroderma.

If active capillary loss is occurring, there should be angiogenesis and vessel replacement in the superficial horizontal plexus at the post capillary venule[65] involving endothelial proliferation[66]. The pattern of malformed vessels seen in nail beds is, in fact, reminiscent of patterns of angiogenesis seen with localized over expression of VEGF[67], [68]. Evidence published by others that circulating and local VEGF is increased in scleroderma has led to a dilemma. How can elevated VEGF be correlated with loss of the microvasculature? In the scleroderma dermis we see no evidence of endothelial replication in the superficial horizontal plexus suggesting that there is no angiogenic response but we have not found a good explanation for this conundrum.

A possible mechanism for the return of capillaries, consistent with the effects of HDIT/HCT is that endothelial precursor cells are required for angiogenesis[69]–[71]. An infusion of the CD34-selected hematopoietic cell graft may have therefore contributed to the re-capillarization. Unfortunately at the time of the HDIT/HCT study, samples were not taken to analyze levels of circulating endothelial cells. In future studies it may be an excellent way to confirm that endothelial cells have returned to normal in scleroderma. Alternatively, and in our opinion more likely, HDIT/HCT is immunosuppressive and may be ablating some cell that inhibits endothelial regeneration or produces a factor or factors that inhibit endothelial cell regeneration. HDIT/HCT treatment has been investigated for other autoimmune diseases besides scleroderma including systemic lupus erythematosus and multiple sclerosis. It has been previously reported that there was recovery of CD4+ T cell counts after HDIT/HCT at 2 years after HDIT/HCT. However, sustained responses were observed in 63% of patients at a median of 4 years[72]. Immune recovery after HDIT/HCT is associated with increasing thymic-derived naïve CD4+ T cells with a decrease in memory T cells, an increase in regulatory T cells, and broader clonal diversity than was present before HDIT/HCT[72]–[74] These late immune changes support the conclusion that sustained responses may have resulted from the immunomodulatory effects of HDIT/HCT in addition to the early immunosuppression and depletion of autoreactive T cells. After HDIT/HCT, we observed that those patients who grew back capillaries were different from scleroderma of long duration. The patients who received HDIT/HCT had more capillaries and also lost interferon α expression, and regained all VE cadherin expression, whereas the scleroderma in late stages did not. The interferon expression and CD123+cells are interesting in this context since one of the functions of the plasmacytoid dendritic cell is the breaking of tolerance[37], and it may be associated with some of the changes in T cell populations. The high levels of interferon α support the hypothesis that plasmacytoid dendritic cells may be part of the pathogenesis of scleroderma. Interferon α is antiproliferative *in vivo* [75]. The high local levels of interferon α in scleroderma skin may therefore be preventing the formation of new capillaries directly.

In conclusion we propose that rarefaction is an early and possibly “pre-diagnosis” event in scleroderma. This capillary loss is associated with a change in endothelial phenotype of the remaining vessels that has not been previously described. The phenotype of these residual vascular structures appears to be mildly inflammatory but not apoptotic, with no sign of endothelial replication or replacement. We hypothesize that the presence of interferon α, the loss of VE cadherin and the presence of *RGS5* are involved in the process which appears to keep the vessels static and produce fibrosis in this poorly understood disease. We report regeneration of capillaries and resolution of the non-angiogenic phenotype after HDIT/HCT. Our studies were somewhat limited by the retrospective nature, however, future planned studies of this autologous protocol and an allogeneic protocol will include capillaroscopic, ultrasound and MRI microvascular assessment in a subset of patients in the hope of defining these microvascular changes and assessing clinical importance. Regulation of VE cadherin expression, function of *RGS5* producing smooth muscle, appearance of interferon α at other sites of vascular rarefaction, and the apparent ablation by HDIT/HCT of some cell involved in the inhibition of angiogenesis are obviously intriguing targets for continued study. Rarefaction itself is not well studied in the known examples of cardiac failure, hypertension and regression of granulation tissue. Our studies may provide new clues to the more general problem of capillary loss.

## Methods

### Patients in the study

All tissues used in this study were biopsies supplied in collaboration with Stanford University, the University of California San Francisco, Boston University, Fred Hutchinson Cancer Research Center, and the University of Washington, collected and analyzed according to Institutional Review Board approved protocols for human study at each respective institution. Normal controls (Table 6) and scleroderma patient biopsies (Table 7) were supplied as sets of formalin fixed paraffin embedded blocks or slides.

**Table 6:** Age, sex and anatomic location of skin biopsies from normal controls

Patient#	Age	Sex	Biopsy Location
1	un	un	scalp
2	un	un	un
3	un	un	scalp
4	45	F	forearm
5	un	un	un
6	un	un	scalp
7	un	un	scalp
8	un	un	breast
9	un	un	abdomen
10	un	un	breast
11	50	F	forearm
12	46	F	forearm
13	51	F	forearm
14	41	M	forearm
15	41	F	forearm
16	64	F	forearm
17	36	M	forearm
18	40	F	forearm
19	59	F	arm
20	47	F	posterior shoulder
21	73	F	lateral neck
22	37	F	arm
23	54	F	posterior neck
24	un	un	thigh

Abbreviations: un-unavailable

**Table 7:** Age, sex, disease subtype and duration and anatomic site of skin biopsies of scleroderma patients

Patient#	Age	Sex	Disease Type	Biopsy Location	Disease Duration
1a	47	F	Diffuse	Forearm	1 year
1b	48			“	2 years
1c	51			“	5 years
2a	44	F	Diffuse	Forearm	1 year
2b	46			“	3 years
2c	“			Back	“
2d	48			Forearm	5 years
3	47	M	Diffuse	Forearm	1 year
4a	44	F	Diffuse	Forearm	1 year
4b	“			Upper Arm	1 year
5	un	M	Diffuse	un	un
6	51	F	Diffuse	Forearm	1 year
7	33	M	Diffuse	Forearm	1.5 years
8	35	M	Diffuse	Forearm	1&emsp14;year
9	44	M	Diffuse	Forearm	6 months
10	28	F	Diffuse	Forearm	2.5 years
11	71	F	Diffuse	Forearm	6 months
12	33	F	Diffuse	Forearm	1.5 years
13	56	F	Diffuse	Forearm	8 months
14	46	F	Diffuse	Forearm	8 months
15	32	F	Diffuse	Forearm	10 months
16	44	F	Diffuse	Forearm	7 months
17	47	F	Diffuse	Forearm	2.5 years
18	39	F	Diffuse	Forearm	6 years
19	55	F	Diffuse	Forearm	9 months
20	40	M	Diffuse	Forearm	1&emsp14;year
21	44	F	Diffuse	Forearm	6 months
22	45	F	Diffuse	Forearm	5 years
23	un	un	Diffuse	Upper Arm	un
24	43	F	Diffuse	Forearm	15 months
25	37	F	Diffuse	Upper arm	7 months
26	50	F	Diffuse	Forearm	1&emsp14;year
27	46	F	Diffuse	Upper arm	11 months
28	40	F	Diffuse	Forearm	2 years
29	55	F	Diffuse	Forearm	2 years
30	36	M	Diffuse	Thigh	15 months

Abbreviations un = unavailable at this time

### HDIT/HCT

The clinical trial of HDIT/HCT for patients with scleroderma has been previously described[19]. Briefly, hematopoietic stem cells were mobilized with granulocyte colony stimulating factor, harvested from the peripheral blood by leukapheresis and then CD34-selected. HDIT/HCT consisted of total body irradiation (800 cGy) with partial lung shielding, high-dose cyclophosphamide (120 mg/kg) and antithymocyte globulin (Pfizer Inc., New York, NY). After HDIT/HCT, patients were transplanted with the autologous CD34-selected hematopoietic cell graft.

### Histochemistry

Tissue was stained with hematoxylin & eosin and Movat's pentachrome according to standard protocols. In addition to these two stains, tissue was stained for endogenous alkaline phosphatase. Briefly, the sections were cut, dewaxed with xylene and rehydrated with ethanol series prior to pretreatment with 100 mM glycine and 0.3% Triton X-100. They were then allowed to develop overnight in 5-Bromo-4-Chloro-3′-Indolyphosphate p-Toluidine Salt/Nitro-Blue Tetrazolium Chloride (Roche). Counterstain is Nuclear Fast Red (Vector Labs). The slides were mounted with Aquamount (VWR Scientific).

### Immunohistochemistry (IHC)

Antibodies are listed in Table 8 with dilution, manufacturer and clone number and secondary antibody.

**Table 8:** Antibodies used for immunohistochemical analysis of skin biopsies.

Antibody	Manufacturer	Clone	Pretreatment	Dilution	Secondary/Kit
Signal trasducer and activator of transcription 1 (STAT1)†	CellSigTech	Tyr701, 58D6	Citrate/heat	1∶200	Biomouse/rabbit Elite
P Selectin (CD62P)†	Abcam	AK-6	Citrate/heat	1∶500	Biomouse/rabbit Elite
P Selectin glycoprotein ligand 1 (PSGL1) †	Abcam	3E2.25.5 PL1	Citrate/heat	1∶200	Biomouse/rabbit Elite
Interleukin-1alpha (IL-1α) †	R&D Systems	Lot #AAB01	Citrate/heat	5 ug/mL	Biomouse/rabbit/goat Elite
Vascular endothelial (VE) Cadherin‡	Novocastra	BV6	Citrate/heat	1∶100	Vector Biomouse Elite
Platelet endothelial cell adhesion molecule 1 (CD31)‡	Novocastra	1A10	EDTA	1∶400	Mouse/Envision
*Ulex europaeus* lectin‡	Vector	N/A	pronase	1∶200	Elite SA (no secondary)
Interleukin 3 receptor subunit alpha (CD123) ‡	DAKO	7G3	PXXIV	1∶250	Mouse/Envision
smooth muscle actin‡	DAKO	1A4	pronase	1∶250	Mouse/Envision
Protease activated receptor 2 (PAR2)‡	Zymed	sam11	none	1∶500	Mouse/Envision
Intercellular adhesion molecule 1 (ICAM1)‡	Chemicon	Wcam1	Citrate/heat	1∶200	Mouse/Envision
Vascular cell adhesion molecule 1 (VCAM1)‡	R&D Systems	BBA19	EDTA	1∶1000	Vector BioGoat/Elite
Ki67 antigen (Ki67)‡	DAKO	MIB-1	Citrate/heat	1∶200	Mouse/Enviision
Cleaved caspase 3‡	Cell Sig Tech	#9661	EDTA	1∶200	Vector BioGoat/Elite
Von Willebrand factor‡	DAKO	M0616	pronase	1∶100	Mouse/Envision

Detection for all IHC performed using 3,3′-diaminobenzadine (DAB).

† In Lab IHC

‡ Phenopath IHC

#### In-lab IHC

Briefly, our protocol for IHC is as follows: paraffin sections were dewaxed and rehydrated prior to antigen retrieval using microwave method in sodium citrate pH 6.0. Slides were placed in citrate buffer and microwaved for 20 minutes on low power, allowed to cool and placed in blocking buffer for 30 minutes, incubated with primary antibody overnight at 4°C, then washed and incubated with biotinylated secondary antibody (Vector Labs Universal horse anti-mouse/rabbit/goat #BA-1300 or Vector Labs Universal horse anti-mouse/rabbit #BA-1400) for 30 minutes. After secondary antibody, the slides were washed and incubated for 30 minutes with avidin biotin complex reagent Blocking serum, and avidin biotin complex reagent were supplied in Vector Elite Universal Kit (Vector Labs #PK6100). The slides were washed again prior to detection with DAB with Nickel Chloride to create a grey-black color (Vector Labs # SK-4100), the slides were then counterstained with methyl green, dehydrated in ethanol series, cleared in xylene and mounted with Permount (Fisher Scientific).

#### Phenopath IHC

Immunohistochemistry done at Phenopath Labs (www.phenopath.com for their established protocols) is indicated in Table 8, along with protocols optimized for each antibody.

### RNA in situ hybridization

The protocol for RNA in situ including construction and amplification of probe was based on the protocol in the Roche DIG application manual[76]. Proteinase K digestion was performed at a concentration of 5 mg/mL for 20 minutes. Probe concentrations were 1 ng/uL and hybridizations were performed at 42°C overnight. Post hybridization washes followed this protocol: low stringency 4×15 minutes at 37°C, then high stringency 2×30 minutes at 55°C. Detection with alkaline phosphatase was performed with anti DIG F_ab_ (Roche) at a dilution of 1∶500 and the chromogen used was Vector Red (Vector Labs).

### RNA Probe generation

Probe for *RGS5* were made from plasmids with a 1.7 kb fragment of human *RGS5* long previously characterized and published by us[40]. A 1.7 kb segment of DNA contaiing *IFNA1* and a 2.2 kb segment for *IFNA2* were amplified from from a human Sanger DNA library clone (#RP11-354P17 GenBank accession # AL353732). The plasmids were subcloned using the Stratgene pPCRScriptamp kit. In vitro transcription was performed with DIG RNA labeling kit (Roche) and RNA was isolated (Agilent low RNA input fluorescent linear amplification kit protocol, 2006 version 4, page 19). Standard controls using sense RNA probes were run at the same concentration as the antisense probe with each experiment and showed no specific signal. Positive and negative tissue controls were included in every experiment as well to confirm signal. Standard blast searches were done for the DNA segments amplified and no major non-specific cross reactivity was found to other human DNA. Blast searches were done against the other interferon genes. Minor similarity found between interferon α genes and *IFNW*. No similarity was found between the interferon α genes and *IFNB* or *IFNG*.

### Scoring methodology

All high power fields are at a magnification of 20×.

#### Endothelial markers

Platelet endothelial cell adhesion molecule 1 (CD31) yielded a predictable staining pattern for all three groups of patients, and was used as a control for the other endothelial markers including von Willebrand factor, vascular endothelial (VE) cadherin, alkaline phosphatase and *Ulex europaeus* lectin. Serial biopsies were stained with all 5 markers and vessels (identified as clusters of cells positive for CD31) were compared. If vessels were found that had CD31 stain but lacked another marker in a serial section, the biopsy was scored as negative for that marker. The observed negative and positive were recorded in 2×2 frequency tables.

#### *RGS5* Stain score

Normal biopsies hybridized with *RGS5* probes showed 0-1 perivascular cells per high power field. Slides that had more than 2 positive cells per high power field were considered positive. Positive slides always had increased perivascular expression, and some also had many cells positive in the dermal matrix. Negative and positive were recorded in 2×2 frequency tables.

#### Interleukin-3 receptor alpha subunit (CD123) (high endothelial venule phenotype), smooth muscle actin and P selectin (CD62P) stain score

Biopsies were examined for presence or absence of lumens of blood vessels with endothelial cells labeling with CD123, smooth muscle actin and CD62P. Biopsies were scanned until positive vessels were located or until entire biopsy had been examined. Biopsies with positive vessels were recorded as positive. Negative and positive were recorded in 2×2 frequency tables.

#### Interferon α score

Biopsies were scored according to the presence or absence of positive stain anywhere in biopsy. Biopsies with any stain color were scored as positive. Negative and positive were recorded in 2×2 frequency tables.

#### Vascular cell adhesion molecule 1 (VCAM1), Intercellular adhesion molecule 1 (ICAM1), interleukin-1 alpha (IL-1α), p selectin glycoprotein ligand 1 (PSGL1), protease activated receptor 2 (PAR2), Ki67 antigen (Ki67), CD123 (plasmacytoid dendritic cells), and cleaved caspase 3 stain scores

Normal skin biopsies were examined and cells per high power field were quantified. The mean of cells per high power field was calculated and we assumed a normal distribution. We added two standard deviations above the positive range and used that number as a cutoff. Biopsies with an average number of cells higher than the cutoff were scored as positive. Biopsies with an average number of cells lower than the cutoff were scored as negative. Negative and positive were recorded in 2×2 frequency tables.

#### Signal transducer and activator of transcription 1 (STAT1) stain score

Cells with a nuclear stain were considered positive. The numbers of positive cells were counted in 3 high power fields and averaged. The normal skin biopsy range was determined and 2 standard deviations were added to the top of the range for a positive cutoff. Only biopsies with an average number of stained nuclei per high power field greater than the cutoff were considered positive. Negative and positive were recorded in 2×2 frequency tables.

### Capillary counts

For quantification purposes, we have used site-matched skin biopsies, since capillary loops change in number from site to site. Biopsies of normal, scleroderma and disease controls were stained with CD31. Photographs of entire biopsy to be counted were taken at 20× with an Olympus microscope and Spot camera. Two separate blinded investigators counted the same set of photographs and data are averages of the two counts. “Profiles” are defined as CD31 stained vessels without a central lumen “Lumens” are defined as CD31 stained vessels with central lumens. “Vessels” are defined as the sum of lumens and profiles.

### Statistics

The stain scores for IHC and RNA *in situ* hybridization were recorded as frequency data and were analyzed for significance using Fishers Exact Significance test. Frequency data were obtained according to above scoring systems and 2×2 tables were used to record differences between groups. P values were calculated with one tailed probability value. Capillary quantification is a continuous variable and was therefore tested for significance using the students' t test for independent variables. Values of p<0.05 were considered significant. Standard error was calculated with the standard deviation divided by the sq root of the N.

## References

[1] Jimenez SA, Hitraya E, Varga J (1996). Pathogenesis of scleroderma. Collagen.. Rheum Dis Clin North Am.

[2] LeRoy EC (1996). Systemic sclerosis. A vascular perspective.. Rheum Dis Clin North Am.

[3] Sapadin AN, Esser AC, Fleischmajer R (2001). Immunopathogenesis of scleroderma–evolving concepts.. Mt Sinai J Med.

[4] Handa R, Kumar U, Pandey RM, Aggarwal P, Biswas A, Wali JP (2002). Raynaud's Phenomenon-A Prospective Study.. J Ind Acad Clin Med.

[5] Kahaleh MB (2004). Vascular involvement in systemic sclerosis (SSc).. Clin Exp Rheumatol.

[6] Jones NF, Clements PJ, Furst DE (2003). Surgical Treatment of the Hand in Systemic Sclerosis.. Systemic Sclerosis.

[7] Carpentier PH, Maricq HR (1990). Microvasculature in systemic sclerosis.. Rheum Dis Clin North Am.

[8] Larcher F, Murillas R, Bolontrade M, Conti C, Jorcano JL (1998). VEGF/VPF overexpression in skin of transgenic mice induces angiogenesis, vascular hyperpermeability and accelerated tumor development.. Oncogene Volume.

[9] Isner JM (2000). Tissue responses to ischemia: local and remote responses for preserving perfusion of ischemic muscle.. J Clin Invest Volume.

[10] Detmar M, Brown L, Schön M, Elicker B, Velasco P (1998). Increased Microvacular density and enhanced leukocyte rolling in the skin of VEGF transgeinic mice.. J Invest Dermatol.

[11] Choi JJ, Min DJ, Cho ML, Min SY, Kim SJ (2003). Elevated vascular endothelial growth factor in systemic sclerosis.. J Rheumatol.

[12] Shore AC (2000). Capillaroscopy and assestment of capillary pressure.. British Journal of Clinical Pharmacology.

[13] Houben AJ, Beljaars JH, Hofstra L, Kroon AA, De Leeuw PW (2003). Microvascular abnormalities in chronic heart failure: a cross-sectional analysis.. Microcirculation.

[14] Antonios TF, Singer DR, Markandu ND, Mortimer PS, MacGregor GA (1999). Rarefaction of skin capillaries in borderline essential hypertension suggests an early structural abnormality.. Hypertension.

[15] Denton CP, Black CM, Clements PJ, Furst DE (2003). Pulmonary Vascular Involvement in Systemic Sclerosis.. Systemic Sclerosis.

[16] White B, Clements PJ, Furst DE (2003). Pulmonary Fibrosis in Systemic Sclerosis.. Systemic Sclerosis.

[17] Herrick AL, Clements PJ, Furst DE (2003). Nervous System Involvement in Systemic Sclerosis.. Systemic Sclerosis.

[18] McSweeney PA, Nash RA, Sullivan KM, Storek J, Crofford LJ (2002). High-dose immunosuppressive therapy for severe systemic sclerosis: initial outcomes.. Blood.

[19] Nash RA, McSweeney PA, Crofford LJ, Abidi M, Chen C (2007). High-dose immunosuppressive therapy and autologous hematopoietic cell transplantation for severe systemic sclerosis: long-term follow-up of the U.S. multicenter pilot study.. Blood.

[20] Nash RA, McSweeney PA, Nelson JL, Wener M, Georges GE (2006). Allogeneic marrow transplantation in patients with severe systemic sclerosis: resolution of dermal fibrosis.. Arthritis Rheum.

[21] Garlanda C, Dejana E (1997). Heterogeneity of endothelial cells. Specific markers.. Arterioscler Thromb Vasc Biol.

[22] Newman PJ, Hillery CA, Albrecht R, Parise LV, Berndt MC (1992). Activation-dependent changes in human platelet PECAM-1: Phosphorylation, cytoskeletal association, and surface membrane redistribution.. J Cell Biol.

[23] Higgins JC, Eady RA (1981). Human dermal microvasculature: II. Enzyme histochemical and cytochemical study.. Br J Dermatol.

[24] Duijvestijn AM, Horst E, Pals ST, Rouse BN, Steere AC (1988). High endothelial differentiation in human lymphoid and inflammatory tissues defined by monoclonal antibody HECA-452.. Am J Pathol.

[25] Girard JP, Springer TA (1995). High endothelial venules (HEVs): specialized endothelium for lymphocyte migration.. Immunol Today.

[26] Salomon RN, Hughes CC, Schoen FJ, Payne DD, Pober JS (1991). Human coronary transplantation-associated arteriosclerosis. Evidence for a chronic immune reaction to activated graft endothelial cells.. Am J Pathol.

[27] Romero LI, Zhang DN, Herron GS, Karasek MA (1997). Interleukin-1 induces major phenotypic changes in human skin microvascular endothelial cells.. J Cell Physiol.

[28] Gruschwitz M, von den DP, Kellner I, Hornstein OP, Sterry W (1992). Expression of adhesion proteins involved in cell-cell and cell-matrix interactions in the skin of patients with progressive systemic sclerosis.. J Am Acad Dermatol.

[29] Nystedt S, Ramakrishnan V, Sundelin J (1996). The proteinase-activated receptor 2 is induced by inflammatory mediators in human endothelial cells. Comparison with the thrombin receptor.. J Biol Chem.

[30] Steinberg SF (2005). The cardiovascular actions of protease-activated receptors.. Mol Pharmacol.

[31] Cederqvist K, Haglund C, Heikkila P, Hollenberg MD, Karikoski R (2005). High expression of pulmonary proteinase-activated receptor 2 in acute and chronic lung injury in preterm infants.. Pediatr Res.

[32] Steinhoff M, Corvera CU, Thoma MS, Kong W, McAlpine BE (1999). Proteinase-activated receptor-2 in human skin: tissue distribution and activation of keratinocytes by mast cell tryptase.. Exp Dermatol.

[33] Akers IA, Parsons M, Hill MR, Hollenberg MD, Sanjar S (2000). Mast cell tryptase stimulates human lung fibroblast proliferation via protease-activated receptor-2.. Am J Physiol Lung Cell Mol Physiol.

[34] D'Andrea MR, Rogahn CJ, Andrade-Gordon P (2000). Localization of protease-activated receptors-1 and -2 in human mast cells: indications for an amplified mast cell degranulation cascade.. Biotech Histochem.

[35] Rivera-Nieves J, Burcin TL, Olson TS, Morris MA, McDuffie M (2006). Critical role of endothelial P-selectin glycoprotein ligand 1 in chronic murine ileitis.. J Exp Med.

[36] Sperandio M, Smith ML, Forlow SB, Olson TS, Xia L (2003). P-selectin glycoprotein ligand-1 mediates L-selectin-dependent leukocyte rolling in venules.. J Exp Med.

[37] Facchetti F, Vermi W, Mason D, Colonna M (2003). The plasmacytoid monocyte/interferon producing cells.. Virchows Arch.

[38] Goodbourn S, Didcock L, Randall R (2000). Interferons: cell signalling, immune modulation, antiviral response and virus countermeasures.. Journal of General Virology.

[39] Wieland T, Mittmann C (2003). Regulators of G-protein signalling: multifunctional proteins with impact on signalling in the cardiovascular system.. Pharmacol Ther.

[40] Adams LD, Geary RL, McManus B, Schwartz SM (2000). A comparison of aorta and vena cava medial message expression by cDNA array analysis identifies a set of 68 consistently differentially expressed genes, all in aortic media.. Circ Res.

[41] Cho H, Kozasa T, Bondjers C, Betsholtz C, Kehrl JH (2003). Pericyte-specific expression of RGS5: implications for PDGF and EDG receptor signaling during vascular maturation.. FASEB J.

[42] Gown AM, Willingham MC (2002). Improved detection of apoptotic cells in archival paraffin sections: immunohistochemistry using antibodies to cleaved caspase 3.. J Histochem Cytochem.

[43] Medsger TA, Clements PJ, Furst DE (2003). Classification, Purpose.. Systemic Sclerosis.

[44] Clements PJ, Medsger TA, Feghali CA, Clements PJ, Furst DE (2003). Cutaneous Involvement in Systemic Sclerosis.. Systemic Sclerosis.

[45] Antonios TF, Rattray FM, Singer DR, Markandu ND, Mortimer PS (2003). Rarefaction of skin capillaries in normotensive offspring of individuals with essential hypertension.. Heart.

[46] Braverman IM (2000). The cutaneous microcirculation.. J Investig Dermatol Symp Proc.

[47] Wallez Y, Vilgrain I, Huber P (2006). Angiogenesis: the VE-cadherin switch.. Trends Cardiovasc Med.

[48] Distler O, Distler JH, Scheid A, Acker T, Hirth A (2004). Uncontrolled expression of vascular endothelial growth factor and its receptors leads to insufficient skin angiogenesis in patients with systemic sclerosis.. Circ Res.

[49] Liu X, Zhu S, Wang T, Hummers L, Wigley FM (2005). Paclitaxel Modulates TGFß Signaling in Scleroderma Skin Grafts in Immunodeficient Mice.. PLoS Med.

[50] Singh RK, Gutman M, Bucana CD, Sanchez R, Llansa N (1995). Interferons alpha and beta down-regulate the expression of basic fibroblast growth factor in human carcinomas.. Proc Natl Acad Sci U S A.

[51] Minasian LM, Motzer RJ, Gluck L, Mazumdar M, Vlamis V (1993). Interferon alfa-2a in advanced renal cell carcinoma: treatment results and survival in 159 patients with long-term follow-up.. J Clin Oncol.

[52] Bielenberg D, McCarty MF, Bucana CD, Yuspa SH, Morgan D (1999). Expression of Interferon- is Associated with Growth Arrest of Murine and Human Epidermal Cells.. Journal of Investigative Dermatology.

[53] Beretta L, Caronni M, Vanoli M, Scorza R (2002). Systemic sclerosis after interferon-alfa therapy for myeloproliferative disorders.. Br J Dermatol.

[54] Bachmeyer C, Farge D, Gluckman E, Miclea JM, Aractingi S (1996). Raynaud's phenomenon and digital necrosis induced by interferon-alpha.. Br J Dermatol.

[55] Black CM, Silman AJ, Herrick AI, Denton CP (1999). Interferon-alpha does not improve outcome at one year in patients with diffuse cutaneous scleroderma: results of a randomized, double-blind, placebo-controlled trial.. Arthritis Rheum.

[56] Theofilopoulos AN, Baccala R, Beutler B, Kono D (2004). TYPE I INTERFERONS (a/ß) IN IMMUNITY AND AUTOIMMUNITY.. Annual Review of Immunology.

[57] Wollenberg A, Wagner M, Gunther S, Towarowski A, Tuma E (2002). Plasmacytoid dendritic cells: a new cutaneous dendritic cell subset with distinct role in inflammatory skin diseases.. J Invest Dermatol.

[58] York MR, Nagai T, Mangini AJ, Lemaire R, van Seventer JM (2007). A macrophage marker, Siglec-1, is increased on circulating monocytes in patients with systemic sclerosis and induced by type I interferons and toll-like receptor agonists.. Arthritis Rheum.

[59] Siegal FP, Kadowaki N, Shodell M, Fitzgerald-Bocarsly PA, Shah K (1999). The nature of the principal type 1 interferon-producing cells in human blood.. Science.

[60] Jimenez SA, Derk CT (2004). Following the molecular pathways toward an understanding of the pathogenesis of systemic sclerosis.. Ann Intern Med.

[61] Kuwana M, Okazaki Y, Yasuoka H, Kawakami Y, Ikeda Y (2004). Defective vasculogenesis in systemic sclerosis.. Lancet.

[62] Blann AD, Illingworth K, Jayson MI (1993). Mechanisms of endothelial cell damage in systemic sclerosis and Raynaud's phenomenon.. J Rheumatol.

[63] Renaudineau Y, Grunebaum E, Krause I, Praprotnik S, Revelen R (2001). Anti-endothelial cell antibodies (AECA) in systemic sclerosis–increased sensitivity using different endothelial cell substrates and association with other autoantibodies.. Autoimmunity.

[64] Trotta F, Biagini G, Cenacchi G, Ballardini G, Varotti C (1984). Microvascular changes in progressive systemic sclerosis: immunohistochemical and ultrastructural study.. Clin Exp Rheumatol.

[65] Clough G, Church M, Shepro D, D'Amore PA, Black C, Garcia JG, Granger DN (2005). Vascular Responses in Human Skin.. Microvascular Research; Biology and Pathology.

[66] Gerritsen M, Shepro D (2005). Endothelial Heterogeneity.. MICROVASCULAR RESEARCH: BIOLOGY AND PATHOLOGY.

[67] Senger DR, Van-de WL, Brown LF, Nagy JA, Yeo KT (1993). Vascular permeability factor (VPF, VEGF) in tumor biology.. Cancer Metastasis Rev.

[68] Dvorak HF, Brown LF, Detmar M, Dvorak AM (1995). Vascular permeability factor/vascular endothelial growth factor, microvascular hyperpermeability, and angiogenesis.. Am J Pathol.

[69] Asahara T, Isner JM (2002). Endothelial progenitor cells for vascular regeneration.. J Hematother Stem Cell Res.

[70] Murayama T, Asahara T (2002). Bone marrow-derived endothelial progenitor cells for vascular regeneration.. Curr Opin Mol Ther.

[71] Isner JM, Kalka C, Kawamoto A, Asahara T (2001). Bone marrow as a source of endothelial cells for natural and iatrogenic vascular repair.. Ann N Y Acad Sci.

[72] Storek J, Zhao Z, Lin E, Berger T, McSweeney PA (2004). Recovery from and consequences of severe iatrogenic lymphopenia (induced to treat autoimmune diseases).. Clinical Immunology.

[73] Muraro PA, Douek DC, Packer A, Chung K, Guenaga FJ (2005). Thymic output generates a new and diverse TCR repertoire after autologous stem cell transplantation in multiple sclerosis patients.. J Exp Med.

[74] de Kleer I, Vastert B, Klein M, Teklenburg G, Arkesteijn G (2006). Autologous stem cell transplantation for autoimmunity induces immunologic self-tolerance by reprogramming autoreactive T cells and restoring the CD4+CD25+ immune regulatory network.. Blood.

[75] Angiolillo AL, Sgadari C, Taub DD, Liao F, Farber JM (1995). Human interferon-inducible protein 10 is a potent inhibitor of angiogenesis in vivo.. J Exp Med.

[76] Komminoth P, Grunewald-Jahno S (1996). Detection of mRNA in tissue sections using DIG-labeled RNA and oligonucleotide probes.. Non-Radioactive in Situ Hybridization Application Manual.

