# Correction: Capillary Regeneration in Scleroderma: Stem Cell Therapy Reverses Phenotype?

**DOI:** 10.1371/annotation/6b021f46-17bd-4ffe-a378-a1b8d24a1398

**Published:** 2008-08-18

**Authors:** Jo N. Fleming, Richard A. Nash, D. O. McLeod, David F. Fiorentino, Howard M. Shulman, M. Kari Connolly, Jerry A. Molitor, Gretchen Henstorf, Robert Lafyatis, David K. Pritchard, Lawrence D. Adams, Daniel E. Furst, Stephen M. Schwartz

There was a sentence omitted from the Funding section. The complete funding information is as follows: This work was funded by the scleroderma Research Foundation that had no role in the design and conduct of the study, in the collection, analysis, and interpretation of data, or in the preparation, review and approval of the manuscript. The clinical trial of high-dose immunosuppressive therapy and autologous hematopoietic cell transplantation and the pathology review was funded in part by award N01-AI-05419 from the National Institute of Allergy and Infectious Diseases.

